# Patient involvement in healthcare workers’ practices: how does it operate? A mixed-methods study in a French university hospital

**DOI:** 10.1186/s12913-020-05271-w

**Published:** 2020-05-08

**Authors:** Lucie Malloggi, Brice Leclère, Clément Le Glatin, Leïla Moret

**Affiliations:** 1 Department of Medical Evaluation and Epidemiology, Nantes University Hospital, Hôpital Saint-Jacques, 85 rue Saint-Jacques, 44093 Nantes cedex, France; 2 UMR INSERM 1246 SPHERE MethodS in Patient-centered outcomes and HEalth ResEarch, Universités de Nantes et Tours, Institut de recherche en Santé 2, Nantes, France

**Keywords:** Patient involvement, Patient engagement, Expert patient, Healthcare quality improvement, Hospitals, Mixed-methods

## Abstract

**Background:**

The present challenge for patient involvement is the improvement of healthcare efficiency through a deeper consideration of the patient experience. In hospitals, numerous interventions promoting patient involvement are informally implemented by healthcare workers (HCWs). The first aim of this study was to conduct an overview of hospital HCWs’ experiences of the involvement of patients or their representatives. This overview included the involvement of patients in the domains of healthcare provision and support for other patients, healthcare quality and safety improvement, training and research. The second aim was to describe the challenges and conditions for the development of participative interventions by HCWs.

**Methods:**

We conducted a mixed-methods sequential study at Nantes University Hospital from September 2017 to May 2018. To achieve the first aim, we performed a descriptive analysis of quantitative data collected via a questionnaire survey of 1290 HCWs. To achieve the second aim, we conducted a thematic analysis of qualitative data collected via eight semi-structured interviews with HCWs who reported involving patients or their representatives (family and patient association members) in healthcare.

**Results:**

Among the 213 survey participants (16.5%), 133 reported a total of 424 participative interventions, mostly in the domains of care quality and safety (37%) and care provision and support (29%). The analysis of the qualitative data evidenced three types of factors determining the implementation of such interventions: the profiles of patients and their representatives, the beliefs and attitudes of HCWs, and organisational factors. While leadership from patients and HCWs was a central element in the development of patient involvement interventions, organisations’ capacities to foster a sustainable partnership culture appeared to be the next challenge to promote the patient-as-partner model in health systems. Our results also highlighted numerous benefits of patient and representative involvement for patients and HCWs.

**Conclusions:**

The numerous initiatives reported show that patients and patient representatives participate alongside HCWs in hospitals. It is essential to take into account the facilitating and hindering factors of patient involvement in hospital HCWs’ practices for the further development of current initiatives. Additional studies, especially from the point of view of patients, are needed to complement our findings.

## Background

It is now widely known that patient involvement is a key component for improving healthcare quality by adapting healthcare systems to patients’ needs [1–5]. Alongside increasing evidence from research on the usefulness of engaging patients in their own care towards the development of patient-centred care [6–11], a wider approach has emerged for promoting the involvement of patients and their representatives (PRI), with “representatives” defined as family and patient association members. The patient-as-partner concept is an attempt to overcome limitations of the patient-centred care model to promote genuine patient participation [2, 3, 12]. It is based on the recognition of not only the patient experience but also experiential knowledge and expertise, which contributes to service improvement [2]. This approach suggests a framework for patient participation that incorporates various forms beyond the context of individual care in a continuum of multilevel applications within the healthcare system, as in the Montreal model [2, 3, 12]. Patients and their representatives can thus be involved in healthcare at the macro or strategic level (governance of health policies), the meso or organisational level (design of healthcare services) and the micro or clinical level (peer support) [13]. More broadly, patients can also become involved in other areas of the healthcare system, including research and training. When patients become involved in areas beyond that of their own care for the benefit of the community, their degree of engagement can vary from simple consultation to collaboration and partnership [12].

In France, the emergence of health democracy in the 2000s led to the institutional recognition of new roles for patients. The French national health strategy recently reaffirmed that the contribution of patients and their representatives to designing the French healthcare system is a priority [14]. Although there is a wide diversity of French terms used for patient involvement, depending on the domains, levels and degrees of participation considered, there is no formal consensus on definitions [15]. Patients engaging at a macro level tend to be known as “healthcare user representatives”, while those who become involved at micro and meso levels are often called “expert patients” [16]. This term is commonly used in France to refer to patients and/or family members who draw on their experiential knowledge of coping with chronic conditions to further improve the healthcare system [15, 16]. In hospitals, these patients can take part in healthcare provision for other patients as well as in healthcare organisation and design alongside healthcare workers (HCWs). Expert patients can also engage more broadly in health research [17–21] and training for health professionals, medical students or other patients [22, 23]. PRI at the micro and meso levels is less formalised than at the macro level since healthcare user representatives have gained official status and their participation in hospital governance within commissions is legally required. Expert patients, however, have no official title, which allows them to be involved in a wide range of activities – from consultation to partnership – but creates some difficulties in defining their roles.

It could thus be useful to determine common terminology for expert patient participation [24] to gain a better understanding of the benefits of their contributions [15]. Evaluative research on PRI at the micro and meso levels in hospital settings, including expert patient participation, needs to progress. Research has largely focused on patients’ involvement in their own personal care or in outpatient settings [25–33]. Furthermore, the lack of details on the nature of patient activities and the absence of defined outcomes to assess the impact of interventions suggest that some activities of patients and patient representatives constitute merely token participation [34]. Our intuition was that despite the absence of a structured organisation for this kind of activity, numerous informal PRI interventions are initiated by caregivers at the clinical micro and organisational meso levels as well as in training and health research. One lever to promote PRI initiatives would be to highlight and share information about them among health professionals and decision-makers. Since university hospitals are involved in different domains of care, research and teaching, they provide an ideal setting to assess participation by patients and their representatives in all these aspects.

Based on the Montreal model, the objective of this exploratory study was to provide a first description of the different ways in which PRI is currently implemented in hospital HCWs’ practices. First, the study aimed to describe individual and collective PRI initiatives led by Nantes University Hospital (NUH) HCWs in four domains of their practice: healthcare provision and support for other patients, initial and continuing health professional education and patient training, healthcare quality and safety improvement, and health research. Second, the study aimed to examine the conditions and challenges of PRI at the organisational and clinical levels in hospitals from the HCW viewpoint.

## Methods

### Study design

**Fig. 1:**
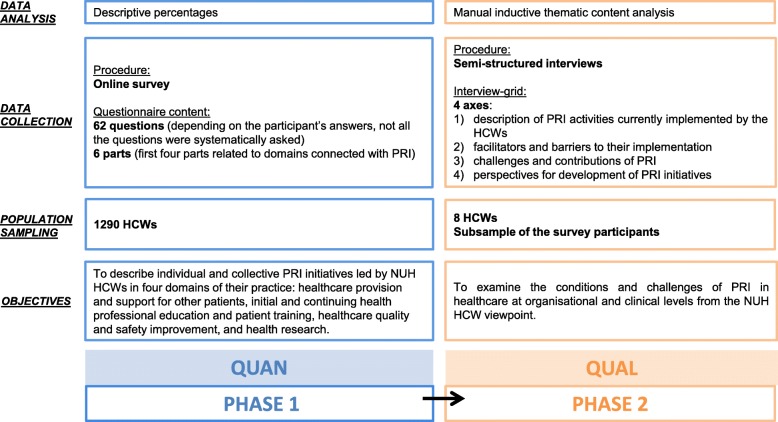
Mixed-methods protocol. HCW: Healthcare Worker. NUH: Nantes University Hospital. PRI: Involvement of Patients and their Representatives

We conducted a two-phase mixed-methods study at NUH from September 2017 to May 2018. This multistrand study employed a sequential design (phase 1: quantitative; phase 2: qualitative) [35–37]. Phase 1 consisted of a descriptive, cross-sectional survey. Phase 2 involved the exploration of qualitative data from in-depth interviews with HCWs. The mixed-methods protocol is summarised in Fig. 1. We used the STROBE guidelines to report quantitative phase 1 and the COREQ guidelines to report qualitative phase 2.

### Reasons for conducting mixed-methods research

Mixed-methods designs are useful to combine several perspectives, providing more comprehensive evidence to answer complex research questions, especially in the fields of public health and health service research [37, 38]. We integrated the quantitative and qualitative data for different conceptual purposes. We endeavoured to ensure *complementarity* [35, 39], as each data type was more closely linked to one of the two aspects of the research question: the quantitative data addressed the first study objective, while the qualitative data addressed the second objective. Another argument for conducting a mixed-methods strategy was *development* [35, 39] since quantitative results were essential to frame the qualitative phase in terms of the sampling and data collection.

### Phase 1 (quantitative)

#### Preliminary assumptions

Phase 1 aimed to describe HCWs’ experiences of PRI in the four domains of their practices. We intentionally avoided a prior definition of what we meant by “involvement” in the questionnaire to obtain a wide range of experiences according to the respondents’ own definitions of PRI practices, thus providing a continuum from consultation to partnership [1].

#### Population sampling

The selection criteria concerned the professions and status of HCWs working at NUH. The eligible population included physicians, dental surgeons, pharmacists, midwives, health executives and nurses specialised in the coordination of therapeutic education programmes who were salaried employees of the hospital. Medical residents and students were excluded. A total of 1290 HCWs working at NUH were selected and invited to participate in an online questionnaire-based survey from September 5th to October 5th, 2017. Participation was voluntary, and completion of the questionnaire was considered informed consent.

#### Data collection

The questionnaire template was created with Sphinx IQ V7.0.2.3® [see Additional file 1]. A single link giving access to the questionnaire was sent from the study coordinator’s (medical doctor working at NUH, last author of this article) professional e-mail address to HCWs’ professional e-mail addresses. We used anonymous professional mailing lists, except for in the nurse sampling. The questionnaire included a total of 62 questions divided into six parts. The first four parts were each related to one domain related to PRI: healthcare provision and support for other patients, health professional education and patient training, healthcare quality and safety improvement, and health research. In each part, a multiple choice introductory question asked the respondent if he/she had the opportunity to involve patients or their representatives in the domain concerned, with response options that corresponded to different types of PRI actions. If the respondent answered positively, he/she was then asked a set of questions focusing on the conditions of the setting of the cited PRI actions. The fifth part of the questionnaire asked the respondents about perceived facilitators and barriers to PRI implementation. As the purpose of this part of the questionnaire was to prepare for data collection during phase 2, the results from these questions will not be presented in this paper. The last part of the questionnaire aimed to collect demographic data.

#### Data analysis

Data were analysed using Microsoft Excel®, R V3.5.3® and R Studio V1.1.463®. The sample characteristics are described as percentages; the sample characteristics were also compared to those of the whole target population using chi-square tests with a 5% α-risk. Qualitative variables are described as percentages. The denominator used to calculate these percentages was the total number of reported PRI actions or the total number of HCWs who reported at least one PRI action.

### Phase 2 (qualitative)

#### Population sampling

The sampled population for phase 2 consisted of a sub-sample of phase 1 participants; purposive sampling was performed based on respondents’ answers and demographic characteristics. The inclusion criteria were having participated in the first-phase survey, having reported at least one PRI action in healthcare and having consented to participate in phase 2 by giving a professional e-mail address at the end of the questionnaire. A total of 14 HCWs, who were chosen to ensure diversity in terms of profession, specialty and working department, were invited by e-mail to participate in the second phase. Ultimately, 8 voluntary HCW participants were included: 5 physicians, 2 nurses and one head nurse. Most of the participating HCWs were women (*n* = 7) and were aged 35 to 55 years old (*n* = 7). They were all from different specialties and hospital departments (haematology (biology), haematology (clinical), infectious diseases, psychiatry, endocrinology, paediatrics, rheumatology, gastroenterology and hepatology).

#### Data collection

Qualitative data were collected through individual face-to-face semi-structured interviews from April to May 2018. The interviewer was the medical public health resident (who presented herself to participants as such) who was responsible for designing the mixed-methods research project and was the first author of this article. The interviewer did not have a previous relationship with any of the participants. Participants knew about the objectives and context of the research when they agreed to participate in the interviews since they had previously participated in the survey and had been re-contacted to take part in the second phase. The duration of the interviews varied from 25 min to 1 h, 16 min. All interviews were audio-recorded, made completely anonymous and transcribed either by the interviewer or by an external provider. We did not perform repeat interviews or seek feedback on the transcripts from the participants. The interview guide [see Additional file 2] was developed based on the quantitative data and the information collected from the open-ended questions from phase 1 to explore these results in more depth. The introductory question was “*Can you tell me how you implement patient participation in your professional practices as an HCW?*”. The remaining questions explored each of the four dimensions reported in Fig. 1.

#### Data analysis

The interviewer manually performed inductive thematic content analysis using Microsoft Excel®. First, she analysed all of the content of each interview, attributing a theme to each meaningful idea, which was then entered into a coding table. Second, she combined the eight coding tables that she had developed to elaborate the coding framework, classifying themes into sections and categories by examining similarities, differences and relationships across the themes. Finally, she prioritised themes and relevant sections for their inclusion in the final coding tree. Data saturation was achieved in the 7th interview and confirmed in the 8th interview. Proper names and some words were replaced with letters in the verbatim transcriptions to ensure confidentiality.

## Results

### Phase 1 (quantitative)

#### Sample characteristics

Among the 1290 HCWs, 213 participated in the survey (16.5% response rate). All of them completed the full questionnaire. Most of the respondents were women, were aged 35 to 55 years old and had a permanent non-university post (Table 1).

**Table 1:** Phase 1: Respondent characteristics and comparison with the population approached

HCW characteristics	Count (percentages)	
	HCWs approached	HCWs included
**Gender** (*p* < 0.001)^a^	*N = 1290*	*N = 213*
**Female**	726 (56%)	156 (73%)
**Age (years)** (*p* < 0.001)^a^	*N = 1290*	*N = 213*
**< 35**	377 (29%)	33 (15%)
**35–55**	656 (51%)	144 (68%)
**> 55**	257 (20%)	29 (14%)
**Unspecified**	–	7 (3%)
**Profession** (*p* = 0.2529)^a^	*N = 1290*	*N = 213*
**Medical profession (physician, dental surgeon, pharmacist, midwife)**	1018 (79%)	146 (69%)
**Head nurse or nurse**	272 (21%)	47 (22%)
**Unspecified**	0	20 (9%)
**Status (if physician, dental surgeon or pharmacist)** (*p* < 0.001)^a^	*N = 931*	*N = 130*
**Permanent university post**	136 (15%)	18 (14%)
**Permanent non-university post**	449 (48%)	86 (66%)
**Non-permanent university post**	110 (12%)	8 (6%)
**Non-permanent non-university post**	236 (25%)	18 (14%)

^a^Chi-square tests: comparison of the HCWs who were approached and those who were included HCWs according to gender, age (“unspecified” category excluded), profession (“unspecified” category excluded) and employment status

*HCW* Healthcare Worker

#### Nature of PRI actions

Two-thirds (*n* = 133) of the respondents reported at least one PRI action in at least one of the four domains (Table 2). In all, 424 PRI actions were reported. PRI actions were most commonly associated with healthcare quality and safety improvement, as this domain accounted for 37% (*n* = 158) of the total number of PRI actions and was cited by 54% (*n* = 72) of the respondents. Almost one-third (*n* = 122) of PRI actions were related to healthcare provision and support for other patients. Involving patients in training and research were mentioned less frequently, as these domains covered 20% (*n* = 85) and 14% (*n* = 59) of the total number of PRI actions, respectively.

**Table 2:** Domains and types of PRI actions cited

Domains and types of PRI actions	Numbers of PRI actions (percentages)	Numbers of HCWs who involved patients in an action in the domain (percentages of HCWs who reported at least one PRI action in at least one of the four domains (*n* = 133))
***Healthcare quality and safety improvement***	***158 (37%)***^***a***^	***72 (54%)***
Organisation and design of care trajectories, integrated care development in inpatient and outpatient settings	64 (41%)^b^	
Patient information or mediation processes between patients and medical teams or hospital administration	40 (25%)^b^	
Care unit organisation, improvement of hospital service delivery	31 (20%)^b^	
Patient safety (medication safety, analysis of serious adverse events, control of care-related infections, etc)	16 (10%)^b^	
Unspecified	7 (4%)^b^	
***Healthcare provision and support for other patients***	***122 (29%)***^***a***^	***80 (60%)***
Development of therapeutic patient education programmes	47 (39%)^b^	
Psychological support for other patients (individual or collective peer support)	40 (33%)^b^	
Entertainment activities for patients in care units	16 (13%)^b^	
Peer support and guidance in patients’ daily lives	16 (13%)^b^	
Unspecified	3 (2%)^b^	
***Initial and continuing health professional education and patient training***	***85 (20%)***^***a***^	***48 (36%)***
Continuing education	37 (44%)^b^	
Medical professions	16	
Paramedical professions	18	
Unspecified	3	
Initial student education	27 (32%)^b^	
Medical studies	12	
Midwifery studies	5	
Nursing studies	5	
Dentistry studies	2	
Pharmacy studies	2	
Physiotherapy studies	1	
Patient training courses	18 (21%)^b^	
Unspecified	3 (3%)^b^	
***Health research***	***59 (14%)***^***a***^	***42 (32%)***
Communication on research projects	25 (42%)^b^	
Identification of research priorities	11 (19%)^b^	
Data collection	8 (14%)^b^	
Design of data collection methods and tools	7 (12%)^b^	
Design of research protocols (research questions, study design, outcomes, etc)	5 (8%)^b^	
Unspecified	3 (5%)^b^	

^a^% of the total number of cited PRI actions (*n* = 424)

^b^% of the total number of PRI actions cited in each domain

*HCW* Healthcare Worker

*PRI* Involvement of Patients and their Representatives

#### Circumstances of the implementation of PRI actions

The circumstances under which the reported PRI actions were implemented are presented in Table 3. Regarding the origins of PRI initiatives, HCWs who involved patients in healthcare mostly declared that these actions originated from the initiative of the hospital department or the care unit or, to a minor extent, from their own personal initiative. Conversely, in the training and research domains, we observed a larger proportion of actions that were initiated by individual HCWs than of actions initiated at the level of a department, service or unit.

**Table 3:** Circumstances of PRI actions in each of the four domains

Conditions	Numbers of HCWs (percentages of the total number of HCWs who reported at least one PRI action in the domain)			
	*Healthcare provision and support for other patients*	*Health professional education and patient training*	*Healthcare quality and safety improvement*	*Health research*
	(*N* = 80)^a^	(*N* = 48)^a^	(*N* = 72)^a^	(*N* = 42)^a^
**Did the action result from**^**b**^				
**personal initiative?**	29 (36%)	28 (58%)	24 (33%)	20 (48%)
**the initiative of the care unit/hospital department/university?**	40 (50%)	23 (48%)	32 (44%)	15 (36%)
**a proposal from an external body or association?**	27 (34%)	8 (17%)	10 (14%)	13 (31%)
**Were the patients involved from a patient association?**				
**Yes**	36 (45%)	20 (42%)	22 (31%)	17 (40%)
**Were the patients’ activities administratively formalised (did patients enter into a contractual agreement to carry out their activities)?**				
**Yes**	29 (36%)	12 (25%)	26 (36%)	16 (38%)
**Did patients undergo specific training to participate in these activities?**				
**Yes or ongoing**	22 (27%)	12 (25%)	8 (11%)	5 (12%)

^a^ Total number of HCWs who reported at least one PRI action in the domain

^b^ Multiple-choice question

*HCW* Healthcare Worker

Almost half of the HCWs who reported involving patients in healthcare provision and support for other patients indicated that they collaborated with patient associations (*n* = 36, 45%). For HCWs who reported PRI actions in the training domain (*n* = 20), the proportion who collaborated with patient associations was 42%; for HCWs who reported PRI actions in the healthcare quality and safety improvement project domain (*n* = 22), this proportion was 31%; and for those who involved patients in health research, this proportion was 40% (*n* = 17).

Regarding administrative status, most of the HCWs noted that the patients engaged voluntarily. Indeed, only one-third of the HCWs who reported PRI actions mentioned the existence of a contract or charter of any sort.

Finally, the vast majority of the HCWs who engaged in PRI actions reported that the patients did not undergo any training for the purpose of the participative activities. The highest proportion was observed for actions related to healthcare provision and support for other patients, for which 27% of the respondents (*n* = 22) reported that patients underwent specific training (therapeutic patient education training courses or university degrees).

### Phase 2 (qualitative)

#### Coding tree

Information derived from the interviews enabled the definition of two main sections to structure the coding tree: (1) determining factors for the implementation of PRI actions and (2) perceived benefits of PRI. Among the determining factors, we distinguished (1.1) the profiles of the patients and their representatives, (1.2) HCWs beliefs and attitudes (1.3), and organisational factors. We classified benefits perceived by HCWs based on whether these benefits concerned (2.1) patients, (2.2) HCWs or (2.3) expert patients. Below, the themes for each main section are presented according to this classification. The coding tree is detailed in Additional file 3, and more complete information is provided in Additional file 4, which presents verbatim transcriptions for each reported theme.

#### Determining factors for the implementation of PRI actions

##### Patient and patient representative profiles

First, the essential determinants of the development of collaborative practices concerned the profiles of patients and their representatives, who needed to meet certain criteria to become involved.

A suitable profile depended on individual characteristics, an essential component being motivation to volunteer. A positive and dynamic attitude, as well as relational skills for oral communication and integration into the healthcare team, was also thought to be essential for partnership.

Regarding the involvement of expert patients in partnerships, concern about the disease was seen as a key element for collaboration, with attention to some specificities concerning patient care trajectories. Indeed, for HCWs, peer contributions through the sharing of experiential knowledge required these patients to have accumulated a sufficiently rich experiential background. In addition, HCWs stressed the fact that involved patients needed to have a well-balanced experience of the disease, having reached the stage of resilience.

For these patients, involvement also required taking a step back from their personal experience to adopt a more universal view of the experience of the disease and not become destabilised when confronted with other patients’ difficulties. Patients and their representatives needed to understand their places and roles within the partnership to ensure good collaboration. Adequate training enabled them to acquire specific skills and to adapt their positions when collaborating. It also helped HCWs legitimise patients’ roles as partners.

##### HCWs’ beliefs and attitudes

Second, implementing partnerships was seen to depend on the beliefs and attitudes of HCWs themselves.

On the one hand, the dissemination of a partnership culture, which was facilitated by teamwork among HCWs and networking habits, was seen as a core element for developing PRI. Furthermore, HCWs reported the importance of experience feedback to strengthen PRI in their practices. Having a positive experience of PRI projects and receiving positive patient feedback encouraged strong adherence to the participative approach and motivated them to develop further initiatives.

On the other hand, some attitudes among HCWs could result in resistance towards better integration of collaborative practices. HCWs mentioned a lack of awareness of the PRI approach. Involving patients within the healthcare team could thus be perceived as disruptive, leading to difficulties for HCWs in managing this new concept. Another component of resistance was the distrust towards the professionalisation of expert patients. The professionalisation of expert patients could be regarded as a threat as it could question HCWs’ professional identities and eventually lead to competition in the context of human resource management perceived as restrictive. The professionalisation of expert patients could also represent a risk of hindering the authenticity of patients’ discourses and positioning. Finally, HCWs reported hospital staff scepticism towards the integration of associations into hospital practices, partly because of pre-existing conflicting positions between the associative and hospital sectors.

##### Organisational factors

Third, according to HCWs, organisational factors accounted for the success of PRI initiatives.

On the one hand, a crucial positive determinant was leadership from different stakeholders at the different levels.

HCWs’ role in encouraging patient involvement was rooted in their motivation to drive partnership-based initiatives, while networking provided an efficient framework for spreading these initiatives. The encouragement of patient involvement was accomplished based on methodological support provided by HCWs acting as peers towards their untrained colleagues. It also relied on expert guidance from a specialised team providing methodological assistance for the implementation of participative projects.

Patients and their representatives were also thought to occupy a central leadership position in driving PRI initiatives. Partnerships with patient associations were seen as playing a core role in PRI interventions. Indeed, patient associations provided financial, logistical and methodological support, allowing HCWs to draw on their expertise.

Institutional leadership was seen as a fundamental condition of PRI development. First, the political leadership of healthcare institutions at the national and local levels was cited as a key element by HCWs due to these leaders’ efforts to drive sustainable formalised policies promoting PRI. Second, strong political commitment from hospital management was seen as essential.

#### On the other hand, HCWs reported organisational barriers to PRI implementation

The first category referred to difficulties recruiting expert patients. HCWs mentioned the complexity of the recruitment process due to patients’ limited availability and the risk of selecting patients with unsuitable psychological profiles.

The second category of organisational barriers concerned difficulties experienced by HCWs in setting up their projects. They mentioned a lack of financial means, including in terms of a lack of funding and uncertainty about financial sustainability. Other challenges included the lack of knowledge about how to involve patients and their representatives, the need for methodological support to ensure the quality and success of partnerships, and the lack of time to devote to participative projects in addition to HCWs’ regular activities.

The third category of barriers was related to a lack of institutional recognition of expert patients’ places and roles. One argument that HCWs voiced was that a lack of a formalised status could hinder participation because of the availability constraints mentioned above since patients participated voluntarily. A lack of institutional recognition also led to difficulties in access to training for patients who were motivated to participate.

#### Perceived benefits of PRI

##### For patients

According to HCWs, other patients could benefit from expert patient involvement, as expert patients acted as models of recovery, providing inspiration and hope. In addition, the involvement of patients and representatives added value to healthcare support thanks to the complementarity of lay and academic expertise.

*“Ultimately, we remain caregivers; even in a therapeutic education session, you are still a caregiver, even if you want to put it aside as much as possible.” (Interview 4).*

The complementarity of expertise enabled better transfer of knowledge and skills, thus improving patient education. Patient and representative contributions to educational support also included the psychosocial counselling they provided, looking beyond the medical perspective to strengthen psychosocial competencies. Promoting PRI was also seen as a means to sustain the complementarity of medical and nonmedical approaches outside hospital settings through the establishment of links with associations so patients could have access to such resources.

##### For HCWs

HCWs also benefited from PRI due to the complementarity of experiential and scientific knowledge. Combining patient knowledge with the HCW clinical perspective enabled them to design and manage care to be more patient-centred. In this respect, the people who were involved in HCWs’ activities were great assets as partners in developing new practices. Offering new prospects and resources, they contributed to driving innovative projects. They also helped to promote projects among institutional decision-makers by supporting their legitimacy. HCWs reported that the implementation of PRI contributed to changing their relationships with patients based on a new care perspective. This could improve the therapeutic alliance:

*“In the end, I felt that the alliance was better when the patient was there.” (Interview 4).*

##### For expert patients

HCWs perceived that for expert patients, collaboration was a meaningful and constructive experience in their own care trajectories.

*“It’s clear that for him – he actually said so – taking part in the group helped his recovery.” (Interview 2).*

For expert patients, discussion of their experience and the transfer of knowledge to other patients contributed to their recovery processes and resilience. According to HCWs, expert patient involvement was also rewarding, as it led to the recognition of the value of experiential knowledge in addition to that of health professional expertise.

## Discussion

### Main results

This study has shown that numerous PRI interventions can be individually or collectively led by HCWs in hospitals. We identified 424 PRI actions conducted by 133 HCWs working at NUH. Most of these participative interventions concerned the healthcare domain. Patients and their representatives were involved in contributing to team reflection about healthcare organisation and integrated care development, patient information and mediation activities, and the improvement of care unit organisation. They also collaborated in the development of therapeutic education programmes or the provision of healthcare support for peers with chronic conditions. PRI actions related to training and health research seemed comparatively under-developed. HCWs mentioned patient profiles and HCW beliefs and attitudes as determining factors in the implementation of PRI interventions in healthcare. In their opinion, cascading leadership involving operational and political stakeholders appeared to be a core element for sustainable PRI development. The HCWs stressed organisational difficulties pertaining to patient recruitment, project establishment and obstacles arising from a lack of institutional recognition of the expert patient role. For HCWs, some of the benefits of PRI were derived from the complementarity of medical and experiential knowledge for the purpose of developing patient-centred care.

### Strengths and weaknesses of the study

This study is the first French study to focus on present-day field experiences of HCWs in hospital settings of the promotion of patient involvement. It aimed to explore little-known, informal PRI practices among HCWs in various professions and medical specialties, and the findings can help to promote patient engagement in a wide range of practical settings. The study offered opportunities for HCWs to highlight initiatives. The large number and diversity of the PRI experiences reported suggest that the exploratory objective of the research was met. Furthermore, participant selection for the qualitative phase based on the answers to the questionnaire resulted in some heterogeneity in the nature of PRI actions reported. Our qualitative results thus cover a wide range of practices with different levels of participation, which enabled the research objective to be addressed from a global perspective, favouring the transferability of the results in various hospital contexts. However, this study has some limitations. Indeed, although phase 1 had an exploratory goal with the aim of producing an inventory of the different PRI actions to be further analysed in phase 2, one limitation of this survey could be a lack of precision. This could first stem from a lack of representativeness of the sample because of the low participation rate, which could indicate that the respondents primarily represented the most heavily invested stakeholders. Second, a lack of precision could result from the possibility for the same PRI actions to be cited several times by different HCWs belonging to the same care team. However, a careful analysis of the answers of HCWs working in the same care units showed that very few of them were likely to report to exactly the same PRI action. Concerning the qualitative results, the absence of triangulation in the analysis could represent a limitation, as only one person performed the analysis.

### Implications

Notably, the facilitators and barriers to PRI evidenced by our qualitative results overlap with previous findings in various contexts of patient engagement [1, 3–5, 11, 16, 21, 30, 31, 34, 40–46]. Some authors have highlighted the relevance of methodological guidance on how to involve patients, with the need to clearly define patients’ roles and responsibilities within partnerships [31, 40, 41]. Although it is thought that patients’ individual characteristics impact PRI processes [1, 34] and that some skills are needed to guarantee the suitability of the people involved [3, 41], a lack of formal criteria for patient recruitment interferes with collaboration [31] and engenders resistance among HCWs [40]. This consideration should provide input for the development of institutional strategies to encourage PRI in hospital settings. Indeed, the importance of institutional executive commitment in promoting a partnership culture and ensuring adequate logistical and methodological resources was a theme that emerged from the qualitative phase and has been widely underlined elsewhere [5, 42, 47]. In this context, the way HCW beliefs and attitudes can either facilitate (when HCWs personally endorse a partnership approach) [16] or hinder participation [30] offers interesting perspectives for the development of PRI. The influence of HCW beliefs and attitudes is related to cultural issues, which originate from HCW training and organisational environmental aspects in which institutional leadership plays a core role [1, 5, 11, 42]. The underlying question of patient and representative status cannot be separated from organisational factors. Previous research has concluded that instituting a status for engaged patients could be an effective way to formalise their roles and accountabilities, ensuring a framework for their collaboration and thus securing partnerships [34, 40]. Additionally, volunteer status could hinder availability [47]. Furthermore, based on the guidance of experienced HCWs speaking for their colleagues [43, 44] (an aspect that the HCWs in our study linked strongly to experience-sharing), a leadership hierarchy from the strategic to the operational level is highlighted as a key mechanism for successful PRI development in the specific context of hospital settings [34].

### Perspectives

Our findings are in line with the triad of key elements suggested by Baker et al. to achieve patient participation [48]. This triad emphasises the importance of the coordinated involvement and consistent training of three protagonists: patients, HCWs and institutional managers. Evidence of the benefits of this approach for participants [4, 31, 46] is encouraging. Our results show that there is potential for acculturation and tool sharing to provide concrete guidance for HCWs on how to successfully manage PRI in various domains, particularly in the research domain [18, 20, 21]. A promising way forward could be to include patient participation as a central theme in medical student education [23], as in the Montreal model since 2011 [3] and in some French universities; research in this domain is growing [22, 49]. The dissemination of experience feedback among protagonists and the development of reliable performance indicators to assess institutional PRI strategies could also be relevant. Qualitative research methods are undoubtedly complementary to quantitative assessment methods in developing indicators, standards and guidelines to monitor and improve PRI in hospitals. Although our study represents a first attempt in this direction, our results need to be completed and confirmed with a wider, multicentre sample. Furthermore, we adapted a questionnaire designed by the École de santé publique de l’Université de Montréal to conduct a complementary observational study in these hospitals in 2018, aiming to explore the macro level of PRI in hospital governance and policy-making processes and to explore efforts to formalise, from the managerial viewpoint, the patient-as-partner approach [2] in hospitals. Although few studies on this topic have been published, this approach is currently developing, and an international comparison of PRI actions and adoption would be of great interest. Thanks to this exploratory study, the NUH is currently structuring its organisation to make the institutional commitment to PRI development a reality.

## Conclusion

This mixed-methods study explored the nature and conditions of PRI in HCW practices in the areas of healthcare delivery, quality improvement, training and research in a university hospital to contribute to the development of the patient partnership model with a bottom-up approach. A complementary qualitative study to address these objectives from patients’ point of view would undoubtedly complement our findings. We strongly believe that the joint commitment of patients and health professionals provides a central force to move forward in the patient-as-partner perspective within healthcare systems.

## Supplementary information

**Additional file 1.** Questionnaire template in original language (phase 1).

**Additional file 2.** Interview guide in original language (phase 2).

**Additional file 3.** Coding tree.

**Additional file 4.** Verbatim transcriptions illustrating the reported results (phase 2).A selection of the verbatim transcriptions is provided in a translated version in Additional file 4. The selection aimed to capture the gist of the interviews rather than every detail of the remarks. The original verbatim transcriptions and a more complete selection can be obtained upon request to the author.

## Data Availability

The datasets used in the current study are available from the corresponding author on reasonable request.

## Abbreviations

HCW: Healthcare worker
NUH: Nantes University Hospital
PRI: Involvement of patients and their representatives
