# Supplementary material for: Patient involvement in healthcare workers’ practices: how does it operate? A mixed-methods study in a French university hospital
Source: BMC Health Serv Res. 2020 May 8;20:391. doi: 10.1186/s12913-020-05271-w (PMC7206773; doi:10.1186/s12913-020-05271-w)
Supplement: Supplementary file 1 — Additional file 1. Questionnaire template in original language (phase 1). [file 12913_2020_5271_MOESM1_ESM.pdf]

Additional file 1: Questionnaire template in original language (phase 1)

Enquête partenariat patient

Vous allez débuter un questionnaire visant à recenser les démarches d'implication de patients ou de leurs représentants par les professionnels de santé au CHU de Nantes.

Il nécessite 3 à 7 minutes pour répondre.

Les questions sont réparties en 5 modules :

- implication des patients dans les soins

- implication des patients dans la formation

- implication des patients dans la qualité et sécurité des soins

- implication des patients dans la recherche

- freins et leviers

Précédent Suivant

Enquête partenariat patient

Implication des patients dans la dispensation et l'accompagnement des soins

1/5

Avez-vous l'occasion d'impliquer des patients, ou leur entourage, dans l'un de ces domaines ?

- ☐ Participation au développement de l'éducation thérapeutique dans vos services pour d'autres patients (évaluation des besoins et attentes en ETP, élaboration de programmes, co-animation d'ateliers avec les professionnels...)
- ☐ Soutien psychologique des autres patients (animation ou participation à des groupes de paroles...)
- ☐ Animation d'activités dans vos services (activités culturelles, distractions...)
- ☐ Accompagnement des autres patients à l'hôpital ou dans leur vie quotidienne
- ☐ NON
- ☐ Autre

Si 'Autre' précisez :

Dans quel type de prise en charge cette activité se déroule-elle ?

- ☐ Hospitalisation conventionnelle / complète
- ☐ Hospitalisation de jour
- ☐ Hospitalisation de semaine
- ☐ Consultation
- ☐ Autre

Si 'Autre' précisez :

En quelle année avez-vous commencé à impliquer les patients ?

S'agit-il ?

- ☐ D'une initiative individuelle de votre part
- ☐ D'un engagement du service ou du pôle
- ☐ D'une démarche proposée par un autre organisme (association, entreprise, laboratoire, fondation...)
- ☐ Vous ne savez pas
- ☐ Autre

Si 'Autre' précisez :

Ces patients sont-ils impliqués via une association de patients ?

- ○ Oui
- ○ Non
- ○ Vous ne savez pas

Quelle est cette association ?

Précédent | Suivant

CENTRE HOSPITALIER UNIVERSITAIRE DE NANTES

Enquête partenariat patient

Implication des patients dans la dispensation et l'accompagnement des soins

1/5

La participation de ces patients est-elle encadrée (charte, lettre de mission, protocole, contrat...) ?

- Oui
- Non
- Vous ne savez pas

Les patients suivent-ils une formation spécifique pour exercer leurs activités (cours, journée de formation, diplôme, séminaire, rencontre inter-intervenant...) ?

- Oui
- C'est en cours de développement
- Non
- Vous ne savez pas

Laquelle ?

Précédent | Suivant

Enquête partenariat patient

Implication des patients dans l'enseignement ou la formation

2/5

Avez-vous l'occasion d'impliquer des patients, ou leur entourage, dans l'un de ces domaines ?

- [ ] Formation initiale des étudiants
- [ ] Formation continue des professionnels (DU, DIU, masters, séminaires, formations dans vos services...)
- [ ] Formation d'autres patients
- [ ] NON
- [ ] Autre

Si 'Autre' précisez :

De quel type de formation initiale s'agit-il ?

- [ ] Formation des étudiants en médecine
- [ ] Formation des étudiants en odontologie
- [ ] Formation des étudiants en maïeutique
- [ ] Formation des étudiants en pharmacie
- [ ] Formation des étudiants en kinésithérapie
- [ ] Formation des étudiants en soins infirmiers
- [ ] Autre

Si 'Autre' précisez :

De quel type de formation continue s'agit-il ?

- [ ] Formation continue du personnel médical
- [ ] Formation continue du personnel paramédical
- [ ] Autre

Si 'Autre' précisez :

Selon quelles modalités s'organise la participation des patients à votre activité de formateur ou d'enseignant ?

- [ ] Aide à la production du contenu pédagogique des enseignements
- [ ] Témoignages de patients lors de cours
- [ ] Animation des enseignements par les patients
- [ ] Monitorat des étudiants par les patients
- [ ] Autre

Si 'Autre' précisez :

En quelle année avez-vous commencé à impliquer les patients ?

Précédent | Suivant

CENTRE HOSPITALIER UNIVERSITAIRE DE NANTES

Enquête partenariat patient

Implication des patients dans l'enseignement ou la formation

2/5

S'agit-il ?

- ☐ D'une initiative individuelle de votre part
- ☐ D'un engagement du service, du pôle ou de la faculté
- ☐ D'une démarche proposée par un autre organisme (association, entreprise, laboratoire, fondation...)
- ☐ Vous ne savez-pas
- ☐ Autre

Si 'Autre' précisez :

Ces patients sont-ils impliqués via une association de patients ?

- ○ Oui
- ○ Non
- ○ Vous ne savez pas

Quelle est cette association ?

La participation de ces patients est-elle encadrée (charte, lettre de mission, protocole, contrat...) ?

- ○ Oui
- ○ Non
- ○ Vous ne savez pas

Les patients suivent-ils une formation spécifique pour exercer leurs activités (cours, journée de formation, diplôme, séminaire, rencontre inter-intervenant...) ?

- ○ Oui
- ○ C'est en cours de développement
- ○ Non
- ○ Vous ne savez pas

Laquelle ?

Précédent | Suivant

Enquête partenariat patient

Implication des patients dans la qualité et la sécurité des soins

3/5

Avez-vous l'occasion d'impliquer des patients, ou leur entourage, dans l'un de ces domaines ?

Communication

- ☐ Médiation entre les patients et les services ou l'administration de l'hôpital (questions, plaintes, doléances...)
- ☐ Information des usagers sur leurs droits
- ☐ NON

Evaluation des pratiques professionnelles

- ☐ Analyse des événements indésirables graves
- ☐ Implication des usagers dans l'analyse des parcours de soins (parcours patients traceurs,...)
- ☐ Réflexion autour de la pertinence des soins ou d'examens
- ☐ NON

Organisation

- ☐ Organisation du service (ergonomie, dotation en matériel, ...)
- ☐ Organisation du parcours de soins des patients dans l'hôpital
- ☐ Développement des réseaux de soins avec la ville
- ☐ NON

Réflexions sur des thématiques spécifiques

- ☐ Hygiène, lutte contre les infections associées aux soins
- ☐ Prestations hospitalières annexes (élaboration des repas, qualité de l'hébergement, accueil des familles...)
- ☐ Sécurité et bon usage des médicaments
- ☐ NON

Autre domaine concernant la qualité et de la sécurité des soins

Précisez

En quelle année avez-vous commencé à impliquer les patients ?

S'agit-il ?

- ☐ D'une initiative individuelle de votre part
- ☐ D'un engagement du service ou du pôle
- ☐ D'une démarche proposée par un autre organisme (association, entreprise, laboratoire, fondation...)
- ☐ Vous ne savez-pas
- ☐ Autre

Si 'Autre' précisez :

Précédent | Suivant

CENTRE HOSPITALIER UNIVERSITAIRE DE NANTES

Enquête partenariat patient

Implication des patients dans la qualité et la sécurité des soins

3/5

Ces patients sont-ils impliqués via une association de patients ?

- Oui
- Non
- Vous ne savez pas

Quelle est cette association ?

La participation de ces patients est-elle encadrée (charte, lettre de mission, protocole, contrat...) ?

- Oui
- Non
- Vous ne savez pas

Les patients suivent-ils une formation spécifique pour exercer leurs activités (cours, journée de formation, diplôme, séminaire, rencontre inter-intervenant...) ?

- Oui
- C'est en cours de développement
- Non
- Vous ne savez pas

Laquelle ?

Précédent | Suivant

CENTRE HOSPITALIER UNIVERSITAIRE DE NANTES

Enquête partenariat patient

Implication des patients dans la recherche

4/5

Avez-vous l'occasion d'impliquer des patients, ou leur entourage, dans l'un de ces domaines ?

- ☐ Proposition des sujets de recherche
- ☐ Participation à l'élaboration des protocoles de recherche (rédaction, relecture, validation...)
- ☐ Participation à la construction des outils d'évaluation / de mesure
- ☐ Participation au recueil des données
- ☐ Communication autour des projets de recherche (présentation du projet, des résultats, publications scientifiques, communications grand public...)
- ☐ NON
- ☐ Autre

Si 'Autre' précisez :

En quelle année avez-vous commencé à impliquer les patients ?

S'agit-il ?

- ☐ D'une initiative individuelle de votre part
- ☐ D'un engagement du service ou du pôle
- ☐ D'une démarche proposée par un autre organisme (association, entreprise, laboratoire, fondation...)
- ☐ Vous ne savez pas
- ☐ Autre

Si 'Autre' précisez :

Ces patients sont-ils impliqués via une association de patients ?

- ○ Oui
- ○ Non
- ○ Vous ne savez pas

Quelle est cette association ?

Précédent | Suivant

Enquête partenariat patient

CENTRE HOSPITALIER UNIVERSITAIRE DE NANTES

Implication des patients dans la recherche

4/5

La participation de ces patients est-elle encadrée (charte, lettre de mission, protocole, contrat...) ?

- Oui
- Non
- Vous ne savez pas

Les patients suivent-ils une formation spécifique pour exercer leurs activités (cours, journée de formation, diplôme, séminaire, rencontre inter-intervenant...) ?

- Oui
- C'est en cours de développement
- Non
- Vous ne savez pas

Laquelle ?

Précédent Suivant

Enquête partenariat patient

Leviers et freins

5/5

Quels LEVIERS avez-vous repérés dans la mise en œuvre de ces démarches d'implication des patients ou de leurs représentants (entourage, représentants d'usagers...) ?

Au contraire, quels FREINS avez-vous rencontrés dans la mise en place de ces démarches ?

Avez-vous d'AUTRES PROJETS d'implication des patients ou de leurs représentants dans les domaines de la dispensation et l'accompagnement des soins pour d'autres patients/ de la recherche/ de l'enseignement/ de la qualité des soins ?

Préciser si "Non"

Précédent | Suivant

CENTRE HOSPITALIER UNIVERSITAIRE DE NANTES

Enquête partenariat patient

Freins et perspectives

5/5

Quels sont les FREINS qui vous empêchent d'impliquer des patients dans votre activité ?

Freins structurels

- ☐ Opposition de vos collègues
- ☐ Opposition de votre hiérarchie
- ☐ Manque de soutien de vos collègues
- ☐ Manque de soutien de votre hiérarchie
- ☐ Manque de moyens financiers, matériels ou logistiques
- ☐ Manque de temps

Freins liés aux patients

- ☐ Manque de compétences des patients ou de leurs représentants
- ☐ Incapacité physique ou cognitive des patients
- ☐ Manque d'intérêt des patients pour de telles démarches

Autres freins

- ☐ Mauvaise expérience ou échec précédent
- ☐ Pas d'intérêt pour vous de telles démarches
- ☐ Autre

Si 'Autre' précisez : ____________________

Avez-vous des PROJETS d'implication des patients, ou de leurs représentants, dans les domaines de la dispensation et l'accompagnement des soins pour d'autres patients/ de la recherche/ de l'enseignement/ de la qualité des soins ?

____

Préciser si "Non"

Précédent | Suivant

Enquête partenariat patient

CENTRE HOSPITALIER UNIVERSITAIRE DE NANTES

Le concept partenaire patient

Parmi les termes suivants, lesquels utilisez-vous dans votre pratique courante ?

☐ ☐ ☐
☐ ☐ ☐
☐ ☐ ☐
☐ ☐

Comment avez-vous eu connaissance de ces termes ?

☐
☐
☐
☐
☐

Si 'Autre' précisez :

Précédent | Suivant

CENTRE HOSPITALIER UNIVERSITAIRE DE NANTES

Enquête partenariat patient

Données socio-démographiques

Dans quel CHU exercez-vous ?

- ○ Rennes
- ○ Nantes

A quel pôle du CHU êtes-vous rattaché ?

[▾]

Si 'Autre' précisez : ______

PHU 1 : Dans quel(s) service(s) exercez-vous ?

- ☐ association du registre des cancers de loire-atlantique
- ☐ blocs opératoires hd
- ☐ clinique chirurgicale digestive et endocrinienne
- ☐ hématologie
- ☐ dermatologie
- ☐ gastro-entérologie-hépatologie et assistance nutritionnelle
- ☐ hémodialyse
- ☐ néphrologie et immunologie clinique
- ☐ oncologie pédiatrique
- ☐ unité médicale ambulatoire de cancérologie
- ☐ urologie
- ☐ Autre

Si 'Autre' précisez : ______

PHU 2 : Dans quel(s) service(s) exercez-vous ?

- ☐ centre de la mémoire
- ☐ chirurgie thoracique et cardiovasculaire
- ☐ chirurgie vasculaire
- ☐ clinique cardiologique et des maladies vasculaires
- ☐ consultations groupées (hgrl)
- ☐ consultations spécialisées (hgrl)
- ☐ endocrinologie
- ☐ exploration fonctionnelles hd
- ☐ explorations fonctionnelles hgrl
- ☐ hémodynamique et radiologie thoracique et vasculaire
- ☐ hôpital de jour mutualisé (institut du thorax)
- ☐ neurologie
- ☐ oncologie thoracique
- ☐ plateforme d'allergologie
- ☐ pneumologie
- ☐ unité de transplantation thoracique
- ☐ Autre

Si 'Autre' précisez : ______

PHU 3 : Dans quel(s) service(s) exercez-vous ?

- ☐ agence de la biomédecine
- ☐ anesthésie - consultations groupées
- ☐ anesthésie - plateau ctcv
- ☐ anesthésie - plateau mère-enfant
- ☐ anesthésie - plateau neurochirurgie-neuroradiologie
- ☐ anesthésie - plateau stont
- ☐ anesthésie - plateau tête et cou
- ☐ anesthésie - plateau urgences-brûlés
- ☐ anesthésie - plateau urologie-digestif
- ☐ anesthésie - hgrl
- ☐ centre fédératif prévention dépistage 44
- ☐ cesu
- ☐ coordination des prélèvements d'organes et de tissus
- ☐ corevih
- ☐ maladies infectieuses et tropicales
- ☐ médecine intensive et réanimation
- ☐ médecine interne
- ☐ Autre

Si 'Autre' précisez : ______

← Précédent | Suivant →

Enquête partenariat patient

Données socio-démographiques

PHU 4 : Dans quel(s) service(s) exercez-vous ?

- ☐ brûlés et chirurgie plastique
- ☐ cellule d'ordonnancement
- ☐ chirurgie maxillo-faciale et stomatologie
- ☐ clinique chirurgicale orthopédique et traumatologique
- ☐ hospitalisation à domicile
- ☐ neurochirurgie
- ☐ neurotraumatologie
- ☐ odontologie
- ☐ ophtalmologie
- ☐ ORL
- ☐ recherche clinique
- ☐ rhumatologie
- ☐ Autre

Si 'Autre' précisez :

PHU 5 : Dans quel(s) service(s) exercez-vous ?

- ☐ biologie et médecine du développement et de la reproduction
- ☐ centre du langage et centre ressource
- ☐ centre Simone Veil de planification familiale
- ☐ chirurgie cardiaque pédiatrique et congénitaux adultes
- ☐ chirurgie infantile
- ☐ cic pédiatrique
- ☐ clinique médicale pédiatrique et consultations pédiatriques
- ☐ équipe mobile douleur pédiatrique
- ☐ gynécologie et obstétrique
- ☐ had pédiatrique
- ☐ hôpital de jour adolescents
- ☐ hôpital de jour médico-chirugical pédiatrique
- ☐ lactarium-diététique
- ☐ oncologie pédiatrique
- ☐ psychologues
- ☐ réanimation pédiatrique et néonatale - néonatalogie
- ☐ réseaux de soins
- ☐ service social des malades
- ☐ unité d'accueil des enfants en danger
- ☐ uhcd pédiatrique
- ☐ unité universitaire de pédo-psychiatrie
- ☐ urgences psychiatriques
- ☐ Autre

Si 'Autre' précisez :

PHU 6 : Dans quel(s) service(s) exercez-vous ?

- ☐ imagerie cardiaque et vasculaire diagnostic
- ☐ médecine nucléaire hgrl
- ☐ médecine nucléaire hd
- ☐ neuroradiologie diagnostique et interventionnelle
- ☐ radiologie centrale hd
- ☐ radiologie générale et abdominale
- ☐ radiologie hôpital mère et enfant
- ☐ Autre

Si 'Autre' précisez :

PHU 7 : Dans quel(s) service(s) exercez-vous ?

- ☐ anatomie cytologie pathologique
- ☐ bactériologie - hygiène hospitalière
- ☐ banque multi-tissu
- ☐ biochimie
- ☐ biothèque
- ☐ centre de prélèvement
- ☐ génétique médicale
- ☐ hématologie (biologie)
- ☐ immunologie
- ☐ parasitologie
- ☐ pharmacologie clinique
- ☐ structures rattachées à la direction du pôle biologie
- ☐ virologie
- ☐ Autre

Si 'Autre' précisez :

Précédent | Suivant

CENTRE HOSPITALIER UNIVERSITAIRE DE NANTES

Enquête partenariat patient

Données socio-démographiques

PHU 8 : Dans quel(s) service(s) exercez-vous ?

- [ ] addictologie et psychiatrie de liaison
- [ ] appartements collectifs
- [ ] CMP La Pérouse
- [ ] CMP Beaumanoir
- [ ] creserc
- [ ] equipad
- [ ] pédopsychiatrie 1 et 2
- [ ] pédopsychiatrie de l'enfant et de l'adolescent
- [ ] pédopsychiatrie de liaison
- [ ] psychiatrie 1
- [ ] psychiatrie 2
- [ ] psychiatrie 3
- [ ] psychiatrie 4
- [ ] psychiatrie 5
- [ ] smpr
- [ ] unité de gérontopsychiatrie
- [ ] unité de gestion des patients en psychiatrie (ugepp)
- [ ] unité de médiations thérapeutique
- [ ] unité universitaire de psychiatrie de l'enfant et de l'adolescent
- [ ] Autre

Si 'Autre' précisez :

PHU 9 : Dans quel(s) service(s) exercez-vous ?

- [ ] consultations gérontologique
- [ ] équipe mobile de gériatrie
- [ ] fonctions transversales (gérontologie)
- [ ] hôpital de jour médecine (gérontologie)
- [ ] médecine aigüe gériatrique
- [ ] médecine polyvalente gériatrique
- [ ] soins de suite et réadaptation gériatrique
- [ ] soins de suite et réadaptation polyvalente
- [ ] soins de suite et réadaptation: unité cognitivo-comportementale
- [ ] unité d'investigation clinique (uic 19)
- [ ] unité de soins longue durée
- [ ] Autre

Si 'Autre' précisez :

PHU 10 : Dans quel(s) service(s) exercez-vous ?

- [ ] médecine du sport et de l'effort physique
- [ ] mpr locomotrice et respiratoire
- [ ] mpr neurologique
- [ ] Autre

Si 'Autre' précisez :

PHU 11 : Dans quel(s) service(s) exercez-vous ?

- [ ] pharmacie
- [ ] service d'évaluation médicale et d'épidémiologie
- [ ] service d'information médicale
- [ ] service de santé au travail
- [ ] service pathologie professionnelle recherche innovation
- [ ] stérilisation
- [ ] Autre

Si 'Autre' précisez :

Précédent | Suivant

CENTRE HOSPITALIER UNIVERSITAIRE DE NANTES

Enquête partenariat patient

Données socio-démographiques

Quelle est votre profession?

- ◯ Médecin
- ◯ Pharmacien
- ◯ Cadre de santé de proximité
- ◯ Maïeuticien(-ne)
- ◯ Directeur de soins
- ◯ Infirmier(-ère) de soins
- ◯ Chirurgien-dentiste
- ◯ Cadre de santé supérieur
- ◯ Autre

Si 'Autre' précisez :

Quel est votre statut ?

- ◯ PU-PH
- ◯ CCA ou AHU
- ◯ Autre
- ◯ MCU-PH
- ◯ Assistant spécialiste
- ◯ PH
- ◯ Praticien attaché

Si 'Autre' précisez :

Vous êtes :

- ◯ Un homme
- ◯ Une femme

Votre tranche d'âge :

- ◯ moins de 35 ans
- ◯ entre 35 et 55 ans
- ◯ plus de 55 ans

Si vous êtes d'accord, merci de nous préciser ici votre ADRESSE MAIL.

Cela nous permettra :

- **de vous transmettre les résultats de notre étude,**
- **éventuellement de vous recontacter pour la réalisation d'un entretien individuel, afin de connaître plus en détails vos pratiques d'implication des patients.**

Si vous le souhaitez, laissez-nous votre avis ou vos commentaires sur cette enquête :

Précédent | Enregistrer
