# Supplementary material for: Patient involvement in healthcare workers’ practices: how does it operate? A mixed-methods study in a French university hospital
Source: BMC Health Serv Res. 2020 May 8;20:391. doi: 10.1186/s12913-020-05271-w (PMC7206773; doi:10.1186/s12913-020-05271-w)
Supplement: Supplementary file 2 — Additional file 2. Interview guide in original language (phase 2). [file 12913_2020_5271_MOESM2_ESM.pdf]

<u>Additional file 2: Interview guide in original language (phase 2)</u>

Introduction

Merci beaucoup de prendre ce temps d'entretien. Votre expérience et votre avis seront très importants pour nous.

Je suis Lucie Malloggi, je suis interne en santé publique dans le service d'épidémiologie et de recherche du CHU. Je vous rencontre aujourd'hui dans le cadre d'une étude que je mène dans le service, avec le Pr Moret et en collaboration avec l'Unité Transversale d'Education Thérapeutique, qui concerne la démocratie en santé et l'implication des patients et de leurs représentants dans le système de santé au CHU. Elle est menée en parallèle au CHU de Rennes.

Cette étude vise à recenser et à décrire les initiatives des professionnels de santé, ayant pour but de faire participer les patients ou leurs proches, et plus largement les usagers du système de santé, dans la dispensation, l'accompagnement et l'organisation des soins, dans l'amélioration de la qualité des soins, dans la formation des professionnels, futurs professionnels et d'autres patients, et dans la recherche. De manière générale, les interventions entreprises se font souvent à l'initiative d'une équipe au sein même des services des établissements de soin, ou dans le cadre du colloque singulier médecin-patient. De fait, souvent informelles, elles sont rarement connues du reste de l'établissement et encore moins des administrations et institutions. Cette étude a pour but de mettre en valeur les initiatives entreprises afin de promouvoir leur développement.

- Une première phase a été réalisée en septembre dernier sous la forme d'une enquête auprès des professionnels de santé susceptibles d'initier, de déployer ou de coordonner dans leurs services des projets répondant à cette volonté de démarche partenariale.
- Les résultats de l'enquête nous ont permis de sélectionner plusieurs professionnels, dont vous faite partie, pour préciser avec eux, au court d'un entretien, les démarches qu'ils ont évoquées dans leurs réponses et mieux comprendre comment ils ont pu les porter.

Il s'agit d'un entretien anonyme et confidentiel. Vous êtes libre de vous exprimer comme vous l'entendez, de ne pas répondre à certaines questions si vous n'en avez pas envie, d'arrêter l'entretien si besoin.

Si vous n'y êtes pas opposé, je vais enregistrer l'entretien pour faciliter l'analyse. Les résultats de cette recherche qualitative seront reportés et présentés de manière totalement anonyme. L'entretien durera environ 45 min.

Avez-vous des questions ? Etes-vous prêt pour commencer ?

Je vous propose, avant de rentrer dans le vif du sujet, de vous présenter et de présenter brièvement vos fonctions et les activités que vous menez au CHU.

**PRESENTATION DE L’ENTRETENU**
-Pouvez-vous présenter en quelques mots les **fonctions et activités professionnelles** que vous menez au CHU ?
**DESCRIPTION DES DEMARCHES PARTICIPATIVES**
-Pouvez-vous me raconter **comment vous impliquez des patients dans votre pratique professionnelle** ?
-Comment cela s’est **mis en place** : comment est **né** le projet et comment il a **pris forme puis évolué** ?
-Volonté **collective (démarche d’équipe dans le service) / initiative individuelle / projet porté par un organisme extérieur/une association** ?
-**Ampleur** ? Nombre de patients impliqués aujourd’hui ?
-**Recrutement/sollicitation des patients** ? **Statut** de ces patients ?
-Quelle **équipe/quels professionnels/collègues impliqués** dans ces activités ?
-**Collaboration avec des associations** de patients **: comment ? Bénéfices et difficultés** de cette collaboration ?
**FREINS ET LEVIERS**
-**Difficultés rencontrées** dans la mise en place des projets ?<br>- Source de **tensions ? Lesquelles ? Pourquoi selon vous?**
- **Stratégies pour surmonter ces difficultés** ? Qui/qu’est-ce-qui a aidé ?
- Difficultés rencontrées **aujourd’hui ? Pourquoi ? Comment les surmonter ?**
**OUVERTURE: Autres domaines/modalités d’implication non évoquées**
-D’**autres approches participatives** sont-elles mises en place par le professionnel **dans d’autres domaines** ?<br>-SI NON : **Pourquoi ?**
**APPORTS POUR LA PRATIQUE**
-**Pourquoi est-ce important pour vous** de faire participer les patients ? **Quels enjeux percevez-vous ?**
-**Que retirez-vous, personnellement**, de la mise en place des démarches dont vous m’avez parlées ?<br>- Cela a-t-il eu une **influence sur vos pratiques** de manière plus générale ?<br>-Est-ce que cela a **modifié votre vision de votre métier/ de votre fonction ? Comment ?**
-Est-ce que cela a eu des **effets auprès des autres professionnels du service** ? Lesquels ?<br>-Est-ce que cela a **amené au développement de projets participatifs par vos collègues ou confrères** ? Lesquels ?
**AVENIR DES DEMARCHES PARTICIPATIVES**
- Savez- vous si d’**autres projets participatifs en lien avec le service** sont ou vont être mis en place?
- Quel **avenir pour ces pratiques** participatives selon vous ? A développer ? **A structurer ?**<br>- Que pensez-vous de **l’idée de contrats/formations pour les patients-partenaires/patients-ressource ?**<br>- Faut-il selon vous plus de **transparence** dans la mise en place des démarches d’implication ? Que pensez-vous dans ce cadre de clarifier le **statut de ces patients**, qu’ils soient identifiés administrativement par les institutions pour reconnaitre leurs compétences comme « professionnelles » ?
**CONCLUSION**
-En quelques mots et pour conclure, **quel sens donnez-vous personnellement à la notion d’impliquer des patients dans sa pratique de professionnel de santé** ?
-Est-ce que vous auriez des **choses à ajouter** à cet entretien ? D’autres choses que vous auriez envie d’évoquer avant de terminer?
