# Supplementary material for: Patient involvement in healthcare workers’ practices: how does it operate? A mixed-methods study in a French university hospital
Source: BMC Health Serv Res. 2020 May 8;20:391. doi: 10.1186/s12913-020-05271-w (PMC7206773; doi:10.1186/s12913-020-05271-w)
Supplement: Supplementary file 3 — Additional file 3. Coding tree. [file 12913_2020_5271_MOESM3_ESM.pdf]

Additional file 3: Coding tree

CONDITIONS AND CHALLENGES FOR INVOLVEMENT OF PATIENTS AND THEIR REPRESENTATIVES IN HEALTHCARE

A — Determining factors for the implementation of PRI actions

- A.1 Profiles of patients and patient representatives
  - A.1.1 Skills and attitudes
    - A.1.1.1
    - A.1.1.2
    - A.1.1.3
    - A.1.1.4
  - A.1.2 Expert patient status regarding the disease
    - A.1.2.1
    - A.1.2.2
    - A.1.2.3
  - A.1.3 Stances
    - A.1.3.1
    - A.1.3.2
    - A.1.3.3
    - A.1.3.4
- A.2 Beliefs and attitudes of HCWs
  - A.2.1 Positive
    - A.2.1.1 Partnership culture
      - A.2.1.1.1
      - A.2.1.1.2
      - A.2.1.1.3
      - A.2.1.1.4
    - A.2.1.2 Positive reinforcement from feedback of experience
      - A.2.1.2.1
      - A.2.1.2.2
  - A.2.2 Negative
    - A.2.2.1 Resistance to change/to partnership approach
      - A.2.2.1.1
      - A.2.2.1.2
      - A.2.2.1.3
        - A.2.2.1.3.1
        - A.2.2.1.3.2
      - A.2.2.1.4
- A.3 Organisational factors
  - A.3.1 Positive
    - A.3.1.1 Leadership
      - A.3.1.1.1 Leadership by HCWs
        - A.3.1.1.1.1
        - A.3.1.1.1.2
        - A.3.1.1.1.3
        - A.3.1.1.1.4
      - A.3.1.1.2 Leadership by patients or representatives
        - A.3.1.1.2.1
        - A.3.1.1.2.2
      - A.3.1.1.3 Institutional and policy leadership
        - A.3.1.1.3.1
        - A.3.1.1.3.2
  - A.3.2 Negative
    - A.3.2.1 Difficulties recruiting expert patients
      - A.3.2.1.1
      - A.3.2.1.2
    - A.3.2.2 Obstacles to implementation
      - A.3.2.2.1
      - A.3.2.2.2
      - A.3.2.2.3
    - A.3.2.3 Lack of institutional recognition of the role of expert patients
      - A.3.2.3.1
      - A.3.2.3.2
        - A.3.2.3.2.1
        - A.3.2.3.2.2

B — Perceived benefits of PRI

- B.1 For patients
  - B.1.1
  - B.1.2
  - B.1.3
  - B.1.4
- B.2 For HCWs
  - B.2.1
  - B.2.2
    - B.2.2.1
    - B.2.2.2
  - B.2.3
- B.3 For expert patients
  - B.3.1
  - B.3.2

HCWs: healthcare workers
PRI: involvement of patients and their representatives

Coding (themes and sub-themes):

A-Determining factors for the implementation of PRI actions

- A.1
  - A.1.1
    - A.1.1.1-motivation to collaborate
    - A.1.1.2-positive and dynamic attitude
    - A.1.1.3-abilities for oral communication and exchange
    - A.1.1.4-ability to integrate into a team
  - A.1.2
    - A.1.2.1-being concerned by the pathology
    - A.1.2.2-long-term experience of the care trajectory reflecting knowledge derived from experience
    - A.1.2.3-balanced attitude towards the care trajectory with the disease (physical and mental dimensions)
  - A.1.3
    - A.1.3.1-distancing of personal experience to reach more universal experience (expert patients)
    - A.1.3.2-distancing of personal experience so as to avoid excessive vulnerability (expert patients)
    - A.1.3.3-knowing one's role as a partner, its place and its limits
    - A.1.3.4-training to adopt the right attitudes
- A.2
  - A.2.1
    - A.2.1.1
      - A.2.1.1.1-strong adherence to partnership approach
      - A.2.1.1.2-values shared with colleagues
      - A.2.1.1.3-teamwork
      - A.2.1.1.4-networking
    - A.2.1.2
      - A.2.1.2.1-positive experiences of patient involvement
      - A.2.1.2.2-positive feedback from patients
  - A.2.2
    - A.2.2.1
      - A.2.2.1.1-little awareness of the partnership culture
      - A.2.2.1.2-undermining of professional practices
      - A.2.2.1.3-fears linked to the professionalisation of expert patients
        - A.2.2.1.3.1-appearance of competition between expert patients and HCWs
        - A.2.2.1.3.2-loss of authenticity of patient discourse
      - A.2.2.1.4-reluctance towards integrating the associative sector into hospitals

- A.3
  - A.3.1
    - A.3.1.1
      - A.3.1.1.1
        - A.3.1.1.1.1-motivation of the HCWs
        - A.3.1.1.1.2-dissemination of practices via networks
        - A.3.1.1.1.3-support by experienced HCWs for their colleagues
        - A.3.1.1.1.4-methodological back-up by qualified team within the facility
      - A.3.1.1.2
        - A.3.1.1.2.1-motivation of the patients and their representatives
        - A.3.1.1.2.2-partnership with associations: impetus and support from patient associations
      - A.3.1.1.3
        - A.3.1.1.3.1-institutional formalisation of the participative approach on national or regional level
        - A.3.1.1.3.2-strategic support from facility management and the hierarchy
  - A.3.2
    - A.3.2.1
      - A.3.2.1.1-restricted availability
      - A.3.2.1.2-certain volunteering expert patient profiles are unsuitable
    - A.3.2.2
      - A.3.2.2.1-funding absent or short-lived
      - A.3.2.2.2-lack of methodological support
      - A.3.2.2.3-professionals lack time
    - A.3.2.3
      - A.3.2.3.1-volunteer status restricts participation
      - A.3.2.3.2-difficulty in accessing training
        - A.3.2.3.2.1-lack of financial support
        - A.3.2.3.2.2-inadequate training programs

B-Perceived benefits of PRI

- B.1
  - B.1.1-the expert patient personifies hope by embodying a recovery model
  - B.1.2-complementary knowledge: contribution of knowledge and tools to complement the medical care approach
  - B.1.3-psycho-social support via the sharing of experiential knowledge
  - B.1.4-creation of links to non-medical resources

B.2

- B.2.1-complementary knowledge: orienting care towards a patient-centred model
- B.2.2-a committed individual is a resource to add value to practices
  - B.2.2.1-creativity and innovation
  - B.2.2.2-support in promoting projects
- B.2.3-changes in the caregiver-patient relationship

B.3

- B.3.1-commitment on behalf of others is a constructive experience in relation to the illness
- B.3.2-recognition and valorisation of experiential knowledge
