# Supplementary material for: Patient involvement in healthcare workers’ practices: how does it operate? A mixed-methods study in a French university hospital
Source: BMC Health Serv Res. 2020 May 8;20:391. doi: 10.1186/s12913-020-05271-w (PMC7206773; doi:10.1186/s12913-020-05271-w)
Supplement: Supplementary file 4 — Additional file 4. Verbatim transcriptions illustrating the reported results (phase 2).A selection of the verbatim transcriptions is provided in a translated version in Additional file 4. The selection aimed to capture the gist of the interviews rather than every detail of the remarks. The original verbatim transcriptions and a more complete selection can be obtained upon request to the author. [file 12913_2020_5271_MOESM4_ESM.pdf]

Additional file 4: Verbatim illustrating reported results (phase 2)

A-DETERMINING FACTORS FOR THE IMPLEMENTATION OF PRI ACTIONS

A.1.1.1-motivation to collaborate

"The person's motivation is what's the most important." (interview 7)

"Anyway you can see that patients want to get involved." (interview 3)

"He had been very active saying, well, for me it's something that was really helpful when you suggested, when you showed me this thing, it… this vulnerability-stress model really helped me to understand and to find that, in the end, we have the power to do something about our illness, and so I'd like to share this, it had shown me something really great." (interview 2)

A.1.1.2-positive and dynamic attitude

"It's true, we're going to take someone who's really quite positive, someone who knows how to lead… The aim is to motivate people to go forward, so people who are likely to be quite positive." (interview 4)

A.1.1.3-abilities for oral communication and exchange

"I think that's it... it's to be able in fact to share views." (interview 8)

"The person needs to be relatively at ease orally to be able to speak out more easily." (interview 4)

"People must have a sense of dialogue" (interview 1)

A.1.1.4- ability to integrate into the healthcare team

"Something happened, I think, and for a about a year now, it's been easy, she [expert patient] 's been coming a lot (…), to meetings, she needed to get used to it, to feel at ease in the team" (interview 2)

"We're really lucky to get on and work well together. Which is not always the case,… in a team where there are interesting individual skills it doesn't always work." (interview 5)

A.1.2.1-being concerned by the pathology

"There's the issue of the pathology: it's important in a TPE [Therapeutic Patient Education] program to be a patient with the particular pathology." (interview 4)

A.1.2.2-long-term experience of the care trajectory reflecting knowledge derived from experience

"Regarding the illness, it's true the patient needs to have gone through a long period of illness to talk about a trajectory, an experience, whether it's on a professional or social level, or about communication about the illness, where the resources are, etc. There must be some kind of experience, so it's true, if a patient has… if he can offer his services but has only two or three years of experience, it's not enough." (interview 4)

"And then, it mustn't be a patient for whom the care trajectory is idyllic, there must be things he can share." (interview 3)

A.1.2.3-balanced attitude towards the care trajectory with the disease (physical and mental dimensions)

"I think it should be a patient who is already some distance from his treatment, because it's true, you are always afraid of a relapse." (interview 3)

"It shouldn't be a patient who's too depressed, a patient for whom nothing is right, when it's all about anger, injustice, incomprehension, doctors are all bad, everything… Well, in my opinion, I think it should be …" (interview 7)

"They sent us a patient who was at the start of his illness and who was still angry. We had an interview. What we do is we carry out interviews with these patients, to try and see what their wishes are, what interests them in all of this. And there it was, this patient was angry, asking lots of questions and saying blah, blah, blah, so it wasn't possible." (interview 4)

A.1.3.1-distancing of personal experience to reach more universal experience (expert patients)

"I think that the person's role is really important here, in sharing his own experience of what he has lived through, but at the same time, he must distance himself from it (…) the right distance, he must be like a carer." (interview 5)

A.1.3.2-distancing of personal experience so as to avoid excessive vulnerability (expert patients)

"We probably have to be careful with certain aspects, I mean, we mustn't cause them any difficulties, due to the fact that they are indeed part of a groups of carers, in relation to their own personal experience; being immersed in a reflection on therapeutic education on F [name of the disease] isn't that going to destabilise them? When they listen to other patients for whom things can get difficult. Well, that's what I mean. This is what we must be careful of, they have to… they have to take a step back, but not too much." (interview 6).

"I think that we shouldn't ignore this kind of thing. It happened to us, once, where these people, in relation to what was being described to them, to experiences of patients who had been treated where it ended badly, we could see that they were toppling over to the other side and they were unable to keep that distance anymore. I think it's important for them too because we have to protect them as well." (interview 3)

A.1.3.3- knowing one's role asa partner, its place and its limits

"Yes, what place do they think they have and what limits, what are they capable of? I think we need to take time to work with these people, I mean, is it just a testimony on one aspect or is it about completely co-managing a program, and how is this done, what roles, etc." (interview 2)

A.1.3.4-training to adopt the right attitudes

"I think there has to be a selection and indeed some training." (interview 3)

"It implies that these people need some training in order to be able to explain, so that it doesn't get out of hand." (interview 8)

"Because if things start to go astray, that can be avoided if the person is trained, so as not to use his own personal case to much…" (interview 1)

"He's not just here to share his experience, he's also here as a carer and educator, so we really need people who are trained." (interview 5)

A.2.1.1.1-strong adherence to partnership approach

"For me, I'm convinced about the need for patients to participate." (interview 4)

A.2.1.1.2-values shared with colleagues

"We have developed reflection on care that is globally common to everyone who works here. So I have never had any worries here regarding the quality of the welcome for V [expert patient]." (interview 2)

"I think that we're really... I think we're all convinced of the usefulness, so I don't think there have been any difficulties, I've never had to fight... " (interview 4)

A.2.1.1.3-teamwork

"Everybody is given the same direction at the start, the team at the start is still the same team... well... more or less the same. But the nurses, well... I've got new nurses who have arrived and I'm their senior. So we have new nurses who… it's all part of it, really, and I think that I'm so convinced about it that I provide inspiration... " (interview 4)

"There's really good synergy, we've been working together for years, but indeed, we would like to create a group of patients internally, patients and relatives who are into all these themes, therapeutic education and quality." (interview 6)

A.2.1.1.4-networking

"Alongside, I am part of the network concerning rare pathologies (…) and I am part of a working group (…) linked to patient associations. So I'm very much aware of the patients' role in rare pathologies and the difficulties in obtaining a diagnosis for rare pathologies, and therefore of the importance of placing them at the centre for good communication." (interview 7)

"In any case, the C [organization name] is a regional centre that oversees all the medical, social and associative organisations that have contact with or treat patients with P [name of the disease]. So, in any case, we end up working with different associations. (…) In this TPE commission, there were people who were already involved in patient therapeutic education for P [name of the disease] in hospitals and informal groups; there were also representatives from social and medical centres who joined in. So, in any case, the aim of the C was to coordinate all the people who work on P, so in fact the associations had the right to be there." (interview 5)

A.2.1.2.1-positive experiences of patient involvement

"If you haven't experienced the need for resource-patients, it's not an issue. Everything was absolutely fine. Then we did have resource-patients, but now we don't anymore, oh! How I'd love to have them again! For me, it's necessary for the patients." (interview 4)

"It's already been a great success, which has been incredible."(interview 7)

A.2.1.2.2-positive feedback from patients

"And then I could also see the benefit, and I'm convinced, even if I was convinced before; the fact that I now realise, that I have had feedback from patients, I'm all the more convinced." (interview 4)

A.2.2.1.1-little awareness of the partnership culture

"After that, there are also going to be limitations, it makes me think that at team level, I mean, what does the team think? After all, it wasn't usual at all a few years ago, it has really evolved, to have a relative or a patient in team." (interview 6).

"But I think it can in fact be really complicated … especially when a treatment has not been understood or managed in that way."(interview 2)

A.2.2.1.2-undermining of professional practices

"In fact, I think it's really complicated in terms of positioning, knowing who's who, the fact that there aren't patients on one side and carers on the other, two sides, carers who care and patients being patients and getting treated, and receiving care, it muddies the waters, it's distressing… it can be very worrying for health professionals I think." (interview 2)

A.2.2.1.3.1-appearance of competition between expert patients and HCWs

"One of the main things is that carers are afraid that in the end there might be job cuts in nursing and new jobs for mediators that are not too costly, because they are paid just above the minimum wage, so the salaries are quite low (…). So there can be a slight feeling of competition." (interview 2)

A.2.2.1.3.2-loss of authenticity of patient discourse

"I'm annoyed at the idea of making professionals out of patients. Because, it's just an idea… they become sales representatives." (interview 1)

"I mean, we make professionals out of patients. Ok, but are they actually still patients?" (interview 1)

A.2.2.1.4-reluctance towards integrating the associative sector into hospitals

"Well, it's true that in the department, at the beginning, it was looked upon with doubt, there was tension about welcoming associations inside the hospital, what place to give them, etc."(interview 5)

A.3.1.1.1.1-motivation of the HCWs

"This was a suggestion I made directly, saying that there was a project for creating a first episode group and if you agree, this young man would be happy to work with us on the development of our program." (interview 2)

"After that, we suggested they could get involved if they wanted in the therapeutic education program that we wanted to implement at the university hospital." (interview 7)

A.3.1.1.1.2-dissemination of practices via networks

"These training courses existed before my time. In fact… these courses were first organised by the people in Tours in the nineties. And then I took over from 2000 … 2005. Today, I'm the one who organises, now that I'm one of the oldest, also compared to my colleagues in Tours who are now younger than me." (interview 1)

"So, as I'm on two different sites, in fact I met the patient in my other activity at the day care hospital; so I made a suggestion to him directly and I came back with this suggestion saying that there was a project for creating a first episode group and that if you agree, this young man would be happy to work with us on the development of the program." (interview 2)

A.3.1.1.1.3-support by experienced HCWs for their colleagues

"When we developed this program on a regional scale, we already had some experience working as a group, when we were co-managing, and because of this, as we were more at ease in group work, it helped other teams at regional level to get started in group work too, supported by our experience." (interview 5)

A.3.1.1.1.4-methodological back-up by a qualified team within the facility

"I really put my forty hours of training [a training course offered by the TPE coordination unit] to good use, plus the ten hours as program coordinator to really get to grips on this. And at the same time, D [a member of the TPE coordination unit staff] says to me, listen, it's really interesting, we love new projects and all, we must organise a patient focus group. We can't organise a therapeutic education project without the patients, without having the patients' expectations." (interview 7)

"This training course [a training course offered by the TPE coordination unit] will help us, and will help the patients understand what their place will be, how they are going to work." (interview 2)

A.3.1.1.2.1-motivation of the patients and their representatives

"It was he [expert patient] who came at the beginning with this project saying "it would be great if we could do something together"." (interview 5)

A.3.1.1.2.2-partnership with associations: impetus and support from patient associations

"Everywhere else, there were compliance consultations but no program, because it was the beginning of compulsory written programs, so there was not always the impetus or the time or the skills to write up a program. And then there was L [name of the patient association] which devised, well, not programs, but patient support (...). So they had substantial experience of this, so in the end, everybody was very happy to be able to make use of this experience." (interview 5)

"The whole framework of this workshop was set up with a patient from R [name of the patient association] with whom we work." (interview 4)

"It is the association that manages everything. It is the association that pays for everything." (interview 1)

"In fact, the idea was that there is a program of quality improvement for this illness in the States, a person named H (...) very much involved in J [name of patient association], thought that it would be interesting, in collaboration with a paediatrician from Y [name of the city] called Z and who has worked on this illness for forty years, and thought it would be a good idea to transfer this American model to France." (interview 6)

A.3.1.1.3.1-institutional formalisation of the participative approach at national or regional level

"So I can't say it was a limitation, in any case it was the wish of ARS [Agence Régionale de Santé] that programs should be written in collaboration with patient associations, it was something extra." (interview 5)

"We gave a presentation of the project in P [name of care unit] at the end of 2016, so the unit had been opened for how long? About ten months; and we went to ARS with our…. just to say hello, to say we exist. Just now we're really small, we would like more resources, etc. but anyway. And in the presentation, we emphasised the fact that we intended to develop peer support, to be able to develop

this within therapeutic education groups and beyond. And the person from ARS who talked to us roughly told us that for the time being they had no resources to help you but they had this project for peer mediation, which is a national project in fact, and that if we agreed, they could, we could... it's experimental funding on a national level." (interview 2)

A.3.1.1.3.2-strategic support from facility management and the hierarchy

"For the time being, I have only discussed things with Mr. N, in the presence of Y the department head, and of R [member of the TPE coordination unit staff], for this to be one of the priorities for our department and the hospital to support this project and fund it for one year so that we can justify our activity and hope to have something from ARS and be able to say, there we are, the thing has been launched and it's working." (interview 7)

"For us, Mr. G [department head] would like the very best, so of course, we get a lot of support from our department head" (interview 4)

A.3.2.1.1-restricted availability

"Limitations come from people's availability." (interview 1)

"One patient came for a long time, and there's another who came too but then she moved to another city, further away, so it became complicated; and then, she started to work full-time so it wasn't possible for her. So, that's how it is, it's more likely to be people who don't live far away, who have a job that's not too time-consuming and whose health allows them to..." (interview 6)

A.3.2.1.2-certain volunteering expert patient profiles are unsuitable

"Some of my own patients volunteered, but the profiles didn't correspond." (interview 4)

"She threw herself wholeheartedly into this thing, but then she told me I couldn't do it anymore, so I told her, resign. It's ok, someone else will take over; at the moment you're being eaten up.... because she was being negatively influenced by negative people who kept coming back, because... well, in networks and so on it's always negative people who keep coming back, isn't it? So she was wondering about things she had never wondered about before." (interview 7)

A.3.2.2.1-funding absent or short-lived

"Funding comes from ARS and at the end of one year and a half, theoretically it's the hospitals that take over. So the outlook isn't clear." (interview 2)

"No, because I think it's a real struggle, trying to develop something new. For the time being, my story scares everyone in fact. When you start something with no resources, I mean with the same resources, you need to find time, and nurses, they need to use up some of their time allocated to other activities to come and work with you, because you are starting from scratch and you don't know if you're going to get funding or not." (interview 7)

A.3.2.2.2-lack of methodological support

"You don't know how things are going to unfold, you don't know where it's going to take place, you don't know …" (interview 7)

"After that, we're confronted with the fact that, oh no! we should have worked on what I said with my two patients who decompensated in a context where we were trying to promote them in a TPE group, it's because we don't know how to do it, we've never learned and then we find out and … So training is going to help us with this, with help from X as well. It's not about psychology, but I think that if we know how, and know the role of an expert patient in a TPE group etc...." (interview 2)

A.3.2.2.3-professionals lack time

"So for the time being, we've decided to do it on Saturdays because of problems with patient availability, medical and paramedical schedules and... After that, I don't know if it will be viable, I just don't know. But at the moment I can't afford to include it in my current activity, so I have to find time elsewhere. Yes, yes, that's right, that's right. So there we go. But having said that, I don't mind starting something by giving up a few Saturdays, but you can't expect me in the long run to work for free either." (interview 7)

A.3.2.3.1-volunteer status restricts participation

"I think it takes away from us a certain… there are other patients who are still very active. Well, among volunteering patients you often have retired people: the patient I'm talking about is of course retired. It's true that if there were funding for the time devoted to TPE… Maybe we'd be able to have patients who are, let's say "working". Yes, if they were paid." (interview 4)

"What is really complicated is that there are loads of people to whom I say: I think that these patients would be great in these groups, but there's no cover for transport expenses, there's no cover of any kind."(interview8)

A.3.2.3.2.1-lack of financial support

"I had...because there's B [training organization] which provides training for resource-patients, I had someone here who wanted to do this training, it actually costs 800 €, and I understand they can't afford it, but we simply don't have the budget either. So I had to tell them, well... sorry but… (interview 5)

A.3.2.3.2.2-inadequate training programs

"I think that the problem is that I don't know what session they go to; the problem is finding themselves as a patient among professionals. So from this point of view, I think that it won't be enough, because certain things can escape them, certain things won't correspond to their expectations. So there it is; I can see that if my patient was in the same forty-hour training session… I think that it would sometimes have been difficult for her to find herself among psychiatrists, psychiatric nurses and with the paediatric department head." (interview 7)

B-PERCEIVED BENEFITS OF PRI

B.1.1-the expert patient personifies hope by embodying a recovery model

“People could see, it was an asset to be able to say “we are ill but we can also continue working, we can carry on with our normal daily lives more or less like anybody else”.” (interview 4)

"It's like saying: You see, they have a profession and we will be able to make normal young adults out of them. People need to project themselves into the future and to show … to show people who have gone through this stage, it’s really good.” (interview 1)

“In fact, they embodied hope in the recovery process, people who had made progress in their recovery.” (interview 2)

B.1.2-complementary knowledge: contributions of knowledge and tools to complement the medical care approach

“I have the whole bibliography and all the theoretical knowledge, but the experience belongs to them, not me.” (interview 7)

“So there were these two dimensions… one of the aspects was… that our goodwill to try not to be the medical expert, etc. was quite insignificant compared to the reality of the horizontal exchanges that took place. We're always in the position of someone who’s got academic knowledge and who… who shares it, while they have other sources of knowledge that they also share.” (interview 2)

“People share both their tools for recovery and their experience” (interview 2)

“We had indeed delivered a message and clearly, and this resource patient probably knew how to deliver this message better than we did - in any case, the patients that were present clearly got the message, it was taken on board, the information was taken on board.” (interview 4)

“It also helps with the patient booklet, to decide on the information, if it can be given to them before hospitalisation so they don’t feel so lost, because they tend to get an enormous amount of information.” (interview 3)

B.1.3-psycho-social support via the sharing of experiential knowledge

“Very quickly, we wanted to involve the patients (…) So, how can I say this… In fact we realised that what was very much a resource in therapeutic education was of course the academic knowledge that patients were able to acquire, but in the end, it was above all the horizontal exchanges that took place inside the group; in the end, it was probably the best thing in terms of self-esteem, in terms of being less lonely…” (interview 2)

“It helps patients to overcome the ordeal.” (interview 8)

B.1.4-creation of links to non-medical resources

"It also creates a link with E [name of patient association]. I mean there are people who are really isolated and are afraid of knocking on the door of the association; there are also the representations linked to E to which not everybody can relate. Once they have this first contact and this link, it helps them to get in touch with an association much more easily." (interview 5)

"It was quite good, not just for the association, we asked him to come and talk about possible resources for the patients, information resources other than the TPE, meaning patient associations. We also wanted him to come and talk about patient associations." (interview 4)

B.2.1-complementary knowledge: orienting care towards a patient-centred model

"We define work themes with them, themes that are their concern." (interview 6)

B.2.2.1-creativity and innovation

"This is why I'm waiting, I hope to find a resource-patient to create something, because I think these patients know what other patients expect." (interview 4)

"She implemented a lot of things, W [expert patient] is someone who knows her limitations, who knows her needs and who… who knows how to ask, which is extremely resourceful for her as much as it is for us." (interview 2)

"And they have loads of ideas, I mean ideas… We often talk about that too, collective intelligence. Yes, the best ideas are found collectively when confronting each other." (interview 6)

B.2.2.2-support in promoting projects

"Patient associations are for me a real… support, in what we can do. I mean, if we come as doctors, we think that it would very good to do this or that, but then people say "OK fair enough but this is a rare pathology, do you think you'll have patients lining up? Do you really think you're contributing something for them? Do you this, do you that, etc.?" Well … not really. But if I come with a patient association and tell the hospital director "we've been given authorisation, we've demonstrated this and that, etc. and I also have a letter from Q [name of patient association] explaining how they are involved, how important they think it is and how they will relay the information, and the existence of this program on their website, as a result, it will bring people in and boost the program", then it's promising." (interview 7)

B.2.3-changes in the caregiver-patient relationship

"We find ourselves on a level footing with patients who are there with us." (interview 2)

"I realised that we could have different relationships from those we were taught." (interview 6)
"It's another outlook for us as well." (interview 4)

B.3.1-commitment on behalf of others is a constructive experience in relation to the illness

"When parents are experiencing an upset in their life as a couple – it's not always easy for couples – it's for us to say "Listen, you're going to care for another family."So they either reject us saying "Listen, we're going to say no because at the moment it's a nightmare, so no.", or they say yes and from then on for them it's very constructive for them to put words on the illness." (interview 1)

"He collaborated with us and he said that it was really a resource for him, in fact." (interview 2)

B.3.2-recognition and valorisation of experiential knowledge

"It is important. It's a recognition, I felt it for all the patients , well the 2-3 patients that were there… the 2 patients that intervened and the patient who volunteered; it was also important for them this position. They knew they had a place, I mean, their experience, not only did the carers acknowledge it, but they took it into account..." (interview 4)
